# Central and Peripheral Inflammation in Mild Cognitive Impairment in the Context of Alzheimer’s Disease

**DOI:** 10.3390/ijms241310523

**Published:** 2023-06-23

**Authors:** Inès Schmidt-Morgenroth, Philippe Michaud, Fabrizio Gasparini, Alexandre Avrameas

**Affiliations:** 1 Novartis Institutes for Biomedical Research (NIBR), Translational Medicine, 4056 Basel, Switzerland; 2 Institut Pascal, Université Clermont Auvergne, CNRS, Clermont Auvergne INP, 63000 Clermont-Ferrand, France

**Keywords:** neuroinflammation, mild cognitive impairment, biomarkers, interleukin-1β, inflammasome

## Abstract

Mild cognitive impairment (MCI) is characterized by an abnormal decline in mental and cognitive function compared with normal cognitive aging. It is an underlying condition of Alzheimer’s disease (AD), an irreversible neurodegenerative disease. In recent years, neuroinflammation has been investigated as a new leading target that contributes to MCI progression into AD. Understanding the mechanism underlying inflammatory processes involved in the early onset of the disease could help find a safe and effective way to diagnose and treat patients. In this article, we assessed over twenty different blood and cerebrospinal fluid (CSF) inflammatory biomarker concentrations with immunoassay methods in patients with MCI (mild cognitive impairment), non-impaired control (NIC), and serum healthy control (HC). We performed group comparisons and analyzed in-group correlations between the biomarkers. We included 107 participants (mean age: 64.7 ± 7.8, women: 58.9%). CSF osteopontin and YKL-40 were significantly increased in the MCI group, whereas serum C-reactive protein and interleukin-6 were significantly higher (*p* < 0.001) in the NIC group compared with the MCI and HC groups. Stronger correlations between interleukin-1β and inflammasome markers were observed in the serum of the MCI group. We confirmed specific inflammatory activation in the central nervous system and interleukin-1β pathway upregulation in the serum of the MCI cohort.

## 1. Introduction

Mild cognitive impairment (MCI) is a translational stage between normal aging and dementia and is defined by minor but abnormal cognitive decline. Symptoms include memory loss, trouble remembering events or words, and unpredictable behavioral changes [1]. The risk of developing MCI is strongly correlated with age and can evolve in various neurological diseases, including Alzheimer’s disease (AD) [2,3]. Hence, MCI has been considered a preclinical stage of AD. Moreover, AD is the most common form of dementia in the world and its prevalence is expected to increase significantly in the coming years. Investigating biochemical factors involved in MCI could help identify patients at risk of AD progression [4]. AD is a major health challenge of the 21st century, as the relationship between biochemical hallmarks, such as amyloid plaques and neurofibrillary tangles, and disease onset and progression remain unclear. Thus, insufficient diagnosis limits appropriate care for patients who are often diagnosed when moderate-to-late onset symptoms start to impact them [5,6]. Additionally, the standard of care mainly includes symptomatic medications as clinical trials have failed to offer a great benefit–risk ratio for patients [7,8,9,10]. Big effort and investment are still needed to fill the gap for effective disease-modifying treatment in order to stop or reduce disease progression at an early stage.

Growing evidence, including on animal models, demonstrated that abnormal deposition of amyloids could trigger the activation of microglia and astrocytes and, therefore, the release of inflammatory mediators [11,12,13,14]. In this context, inflammation has emerged as a new leading target in various diseases, including MCI and AD [15,16,17]. Nonetheless, a chronic immune response could lead to even greater neuronal damage and indirect toxic effect [18,19]. Focusing on neuroinflammation, circulating inflammatory biomarkers can help understand pathophysiological changes associated with the disease, taking place in the brain and the systemic circulation.

In recent years, the development of highly sensitive quantification technologies has allowed the field to broaden its research area to new potential targets for diagnosis or treatment. CSF collection alongside molecular imaging using positron emission tomography (PET) have been used as a reference to monitor amyloids and tau proteins, assuming it would reflect biochemical changes occurring in the central nervous system (CNS). CSF collection, although it has helped gain insight into diagnosis, especially at the early onset of the disease, remains a painful, invasive, and expensive method for patients [20]. Therefore, biomarker investigations were extended to blood biomarkers as they could help find a more effective, less invasive, and painless way to detect the disease [21]. In recent years, several blood and CSF candidates have been examined to be promising for AD diagnosis, including, non-exhaustively, neurodegenerative, brain damage, glial response, and astrocytic biomarkers [22].

Among promising candidates, soluble biomarkers associated with the CNS, mainly secreted by microglia and astrocytes, have been investigated for modulating the inflammatory response, including activation of proinflammatory cytokines production. Biomarkers have been sought to be involved in structural or functional roles of brain resident cells such as neuron and blood–brain barrier (BBB) support, cell migration and communication, and amyloid plaque clearance [23,24,25,26]. Glial fibrillary acidic protein (GFAP), neurofilament light (NFL), and triggering receptor expressed on myeloid cells 2 (TREM-2) are examples of biomarkers that have been reported in AD, mainly for their biological functions in the CNS. However, to comprehend the origin of neuroinflammation, the focus has been expanded to soluble mediators from the blood. Cytokines of general inflammation, including interleukin (IL)-1β, have also been correlated with AD and aging [13,26,27,28,29,30,31]. In addition to its role in general inflammation, IL-1β plays a potential, but controversial, role in neurodegenerative disease development as results remain inconsistent between studies, some supporting increased and some showing unaltered IL-1β levels or expression in Alzheimer patients [27,32,33,34,35].

In the present study, we investigated if patients with mild cognitive impairment had a specific central or peripheral inflammatory signature reflecting the early onset of AD. We compared different soluble biomarkers in the CSF and serum of three populations, one MCI, one non-impaired control (NIC), which consisted of cognitively healthy individuals (mini-mental state examination score of 30) with osteoarthritis, and one healthy control (HC). Furthermore, we wanted to understand the systemic inflammation potential role in AD development and how it could be interconnected with neuroinflammation. Accordingly, we added biomarker measurements of systemic inflammation. To better understand the biological mechanism underlying IL-1β production, we focused here on proteins involved in the NLRP3 (nucleotide-binding oligomerization domain (NOD), leucine-rich repeat, and pyrin domain-containing receptor protein 3) inflammasome pathway. Activation of this pathway promotes the production of cytokines including IL-1β and IL-18 and is involved in pyroptosis, a form of programmed cell death [36]. In Alzheimer’s disease, its activation has been highlighted to affect amyloid and tau deposition through microglia stimulation [37,38].

## 2. Results

The demographic and clinical characteristics are shown in Table 1. We selected 107 participants including 32 with MCI, 45 non-impaired control (NIC), and 30 healthy control (HC). Because CSF collection is only permitted for diseased patients, we selected patients with osteoarthritis, but cognitively unimpaired, as the CSF and serum biomarkers control. We then added a cohort of healthy control, as the peripheral inflammation biomarker control, with only serum available.

Of the 107 included participants, the mean age was 64.7 (7.8) years, and the subjects were mainly women (58.9%). Patients with MCI were older than the NIC and HC subjects 69.1 (8.3) vs. 64.8 (6.5) and 59.6 (6.1), respectively. The difference in age was significantly different between the HC vs. NIC groups and the HC vs. MCI groups but not between the NIC and MCI groups. Most patients with MCI and NIC were women (62.5% and 62.2%, respectively), whereas the HC patients were equal in the number of men and women. A total of 68.8% of MCI patients had heart- or vascular-related concomitant disease (31.3% hypertension and 37.5% ischemic heart disease). Additionally, all NIC subjects suffered from osteoarthritis, being either knee arthritis or a disc herniation, 71.1% and 24.4%, respectively.

Overall, of the twenty-one different biomarkers measured in CSF, six had a very low detection rate or were not quantifiable at all, including IL-1α, IL-1β, IL-1Ra, IL-10, Caspase-1, and tumor necrosis factor (TNF)α as they were below the limits of detection.

In serum, of the twenty biomarkers tested, IL-1α was not quantifiable in any cohort while pTau181 was below the limits of quantification in 53% of the healthy controls only (100% quantifiable in NIC and MCI cohorts).

### 2.1. Comparison of Alzheimer’s Disease Biomarkers: Amyloid and Tau Proteins

First, we investigated the main AD hallmark biomarkers, namely, Aβ42 and Tau proteins, in all three cohorts and in all CSF and serum samples (Table 2). Those proteins are frequently, but not necessarily, assessed with quantitative methods or PET imaging in combination with cognitive evaluation as part of AD clinical diagnostic. We used the INNOTEST (IT) diagnostic test for amyloid β (Aβ) 42, whereas MSD S-plex was used for total Tau (tTau) and phosphorylated Tau (pTau) 181. Beforehand, we compared INNOTEST tTau with MSD S-plex tTau using the NIC CSF samples. To confirm the use of the MSD kit instead of INNOTEST tTau for the rest of the samples, we drew a linear regression and tested the correlation between the two kits: R^2^ = 89% and the *p*-value < 0.0001 (Figure S1 from the Supplementary Materials).

Surprisingly, Aβ42 CSF mean concentration was significantly higher in the MCI group than the NIC group (496.3 pg/mL vs. 735.7 pg/mL; *p* = 0.0002). tTau CSF mean concentration was not significantly different between the two groups: 211.6 pg/mL vs. 198.1 pg/mL for the NIC and MCI populations, respectively. Additionally, no significant difference was observed between the pTau181 CSF mean concentration between the NIC and MCI groups (15.5 pg/mL vs. 16.1 pg/mL, respectively).

Aβ42 and Tau play a critical role in the brain of AD patients, although little is known about their function in the periphery. Consequently, we investigated if serum tau levels would exemplify CSF levels. In the serum, tTau was significantly higher in the NIC cohort compared with the HC cohort (*p* = 0.0002), but not between the HC vs. MCI cohorts (*p* = 0.1561) and the NIC vs. MCI cohorts (*p* = 0.1124). pTau181 was significantly higher in the NIC cohort compared with the MCI cohort (*p* < 0.05). However, we were not able to compare with the HC cohort as pTau181 serum concentration was not detectable for every sample, with only 47% of the samples having concentrations above the limit of quantification. Looking at tTau concentration in absolute values, MCI and NIC were not so different in both matrices and the concentration differences between both cohorts were approximatively the same in CSF and serum.

We applied the Aβ42 CSF cutoff proposed by Hulstaert et al. with the same diagnostic kit, to evaluate if our MCI population was presenting an AD profile or predicting disease development [39]. At this stage, only 25% of MCI patients had positive Alzheimer hallmarks, whereas 62% of the NIC samples were positive. This implied that only one quarter of our MCI cohort would have been considered amyloid positive and potentially AD positive if the diagnosis had been conducted only based on the CSF protein assessment. On the other hand, 62% of the NIC cohort could be considered amyloid positive and potentially AD positive, although they were not affected by cognitive pathological symptoms. At this point, neither Aβ42 nor tau proteins alone were reliable biomarkers to differentiate between the MCI and NIC cohorts.

Reinforcing that the use of soluble Aβ42 and Tau or pTau levels alone as diagnostic biomarkers might not be sufficient, we calculated the pTau181/Aβ42 ratio, as a study found a strong correlation between this ratio and Aβ42 PET imaging [40]. It should be noted that Aβ42 concentration was measured similarly to the one found in the published study; however, the method used to assess pTau181 was different in our case. Hence, this cutoff only provides information on how our populations identify in terms of MCI. The NIC and MCI ratio means were statistically significant between the two groups (*p* = 0.008) but both corresponded to the MCI cohort results published by Harten et al.

Aβ accumulation has been studied as the first driver of AD and as possibly causing inflammation and tauopathy [41]. To evaluate this causal link, we tested the correlations between CSF Aβ42, tTau, and pTau181 in both cohorts (Table S1). In each group, only tTau and pTau181 correlated positively (MCI: r = 0.732, *p* < 0.0001; NIC: r = 0.521, *p* = 0.0013). This corroborates with total Tau measurements, which encompass all post-translational modifications, including phosphorylation.

Comparing the results between CSF and serum (Table S2), no significant correlation was observed in the MCI population between AD biomarkers. In the NIC cohort, comparing biomarkers in serum vs. CSF, pTau181 correlated positively (r = 0.563, *p* = 0.0007). Additionally, we observed a correlation between CSF tTau and serum pTau181 (r = 0.620, *p* < 0.0001).

Overall, comparing CSF AD hallmarks in MCI and NIC cohorts, only Aβ42 was significantly increased in the MCI cohort compared with NIC, whereas CSF tTau and pTau181 levels remained similar. When we applied published cutoffs specific to MCI or AD signatures, they did not correspond with our population characteristics. The results suggest that more NIC individuals presented signature biochemical AD hallmarks than MIC patients. Furthermore, when correlating biomarker concentrations in both matrices, tau and pTau181 correlated positively between CSF and serum in the NIC cohort only and not in the MCI cohort, indicating potentially that blood biomarkers could not be used as CSF surrogates.

### 2.2. Neuroinflammation, Astrogliosis, and Microglia Activation Biomarkers in CSF and Serum

The role of brain resident cells, such as microglia and astrocytes, have been investigated in AD as they are important structural and functional supports to the CNS environment. As a result, the focus has been drawn to secreted mediators, assuming they would reflect CNS cell activation.

The quantified CNS soluble biomarkers are summarized in Table 3. In the CSF, osteopontin (OPN) and YKL-40 (also known as chiniase-3-like1 (CHI3L1)) were significantly increased in the MCI cohort compared with the NIC cohort (*p* < 0.0001 and *p* = 0.0165, respectively). For the rest of the biomarkers, mean concentrations were higher in the MCI cohort, although not significantly. Moreover, a greater heterogeneity was observed for all biomarkers in the MCI cohort.

In the serum, only OPN (*p* < 0.0001) was significantly increased in the NIC cohort compared with the MCI cohort. All other biomarkers were not significantly different between the NIC and MIC cohorts.

Between the MIC and HC cohorts, glial fibrillary acidic protein (GFAP) and tissue inhibitor of metalloproteinase 1 (TIMP-1) levels were both significantly increased in the MCI cohort (*p* < 0.01 and *p* < 0.05, respectively). Soluble triggering receptor expressed on myeloid cells 2 (TREM-2) was the only biomarker significantly higher in the HC cohort compared with the MCI cohort (*p* < 0.05). Compared with the CSF, GFAP and neurofilament light (NFL) protein serum concentrations displayed higher variability in the MCI cohort.

We compared serum vs. CSF results for each biomarker to investigate the potential link between matrices (Table S3). In the MCI cohort, no biomarker had a significant correlation between the CSF and serum. In the NIC cohort, only NFL correlated positively (r = 0.650, *p* < 0.0001) between the two matrices.

To summarize, CSF biomarkers associated with microglia and astrocyte activation were increased in the MCI cohort compared with the NIC cohort, including significantly for OPN and YKL-40, two mediators secreted in the brain and associated with immune cell infiltration and recruitment. On the other hand, most biomarkers had similar serum concentrations between the MCI and NIC cohorts. There were no correlations between biomarker concentrations in the CSF and serum.

### 2.3. Systemic Inflammation Biomarkers

As it is easy to assume that CNS resident cells are activated during AD onset, we wanted to review the influence of the peripheral system and potentially its activation as well. Concentrations of systemic inflammatory biomarkers are compiled in Table 4. Globally, five biomarkers, C-reactive protein (CRP), IL-10, IL-6, monocyte chemoattractant protein 1 (MCP-1), and TNFα were increased in NIC serum. CRP serum mean concentration was significantly higher in the NIC cohort compared with the MCI cohort (12.9 mg/mL vs. 3.72 mg/mL, respectively) and the IL-6 serum mean concentration was significantly higher in the HC and NIC cohorts compared with the MCI cohort (7.81 pg/mL and 42.8 pg/mL vs. 3.1 pg/mL, respectively).

In the MCI cohort, IL-10, MCP-1, and TNFα serum levels were significantly increased compared with the HC cohort, whereas the only biomarker significantly increased in the MCI cohort vs. the NIC cohort was interferon gamma-induced protein 10 (IP-10) (131.7 pg/mL vs. 97.20 pg/mL; *p* < 0.01). Interestingly, IL-8 mean serum concentration was increased as well in the MCI cohort but not significantly (MCI: 87.74 pg/mL vs. NIC: 35.19 pg/mL; *p* = 0.3771).

The NIC cohort displayed a high peripheral inflammatory status, notably with CRP and IL-6 concentrations, which were respectively four and fourteen times higher compared with the MCI population. The MCI inflammatory status was elevated as well; although most biomarker concentrations were not as high as in the NIC cohort, they were still raised compared with the HC population.

### 2.4. Circulating Inflammatory Cytokines in the CNS

Circulating inflammatory mediators are mainly known for their central role in the peripheral system, and some, such as CRP, are routinely measured to evaluate the general inflammatory status of an individual. To explore the potential effect and origin of inflammatory cytokines, we assessed the same biomarkers in the CSF (Table 5). The tendency of most biomarkers was reversed in the CSF as all biomarkers were increased in the MCI cohort, although not significantly. This was particularly striking for CRP and IL-6 concentrations, as both were significantly lower in the MCI cohort serum compared with the NIC cohort; however, CSF was increased.

**Figure 1:**
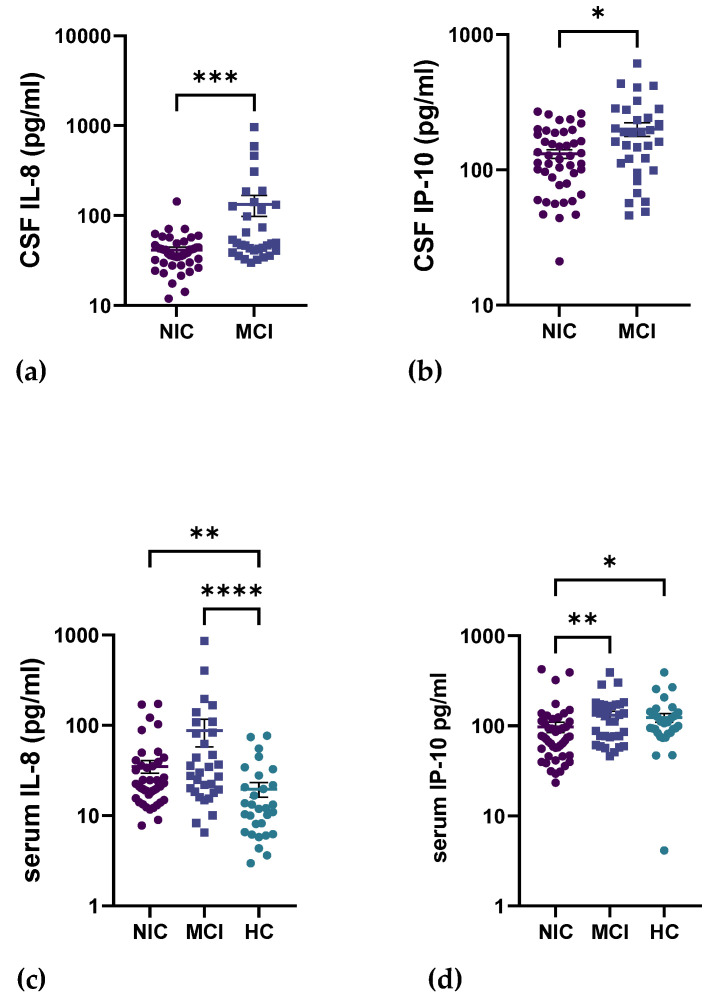
IL-8 and IP-10 scatter plots. Data bars are presented as mean (+/− standard error mean (SEM)). (**a**) IL-8 in CSF; NIC: 41.36 (3.075) pg/mL, MCI: 132.8 (35.02) pg/mL. (**b**) IP-10 in CSF; NIC: 131.4 (9.746) pg/mL, MCI: 199.6 (23.02) pg/mL. (**c**) IL-8 in serum; NIC: 35.19 (5.619) pg/mL, MCI 87.74 (29.57) pg/mL, HC: 19.67 (3.587) pg/mL. (**d**) IP-10 in serum; NIC: 97.20 (12.68) pg/mL, MCI: 131.7 (14.06) pg/mL, HC: 123.4 (13.76) pg/mL. *: *p*-value < 0.05; **: *p*-value < 0.01; ***: *p*-value < 0.001; ****: *p*-value < 0.0001.

Two biomarkers stood out and were significantly higher in the MCI cohort, first IL-8 (MCI: 132.8 pg/mL vs. NIC: 41.36 pg/mL; *p* < 0.001) and IP-10 (MCI: 199.6 pg/mL; NIC: 131.4 pg/mL; *p* < 0.05). Surprisingly, these two biomarkers were the only ones with higher serum concentrations in the MCI subjects as well (Figure 1).

To evaluate if there were any direct links between levels found in serum and CSF, especially for IL-8 and IP-10, we correlated the biomarkers in both matrices. These data are summarized in Table S4. First, comparing IL-8 and IP-10 concentrations in serum vs. CSF, there was no significant correlation in the MCI or NIC cohorts. This was the case for all inflammatory cytokines in the MCI cohort. Looking at the MCI cohort, IL-18 was the only biomarker that correlated positively between the two matrices (r = 0.6151, *p* < 0.0001).

We further compared all CSF vs. serum biomarkers together. In the MCI population, two significant correlations were found, IL-6 correlated positively with CRP and MCP-1 (r = 0.501, *p* = 0.0041, and r = 0.525, *p* = 0.0020, respectively). On the opposite, no significant results were observed in the MCI individuals.

Altogether, the results suggest that inflammatory status in the CNS is higher in the MCI cohort compared with the NIC cohort. There were no direct correlations between the biomarkers in serum and CSF, indicating that the link between the periphery and the CNS is not so straightforward. In fact, it is more likely that inflammatory mediators are independently secreted in both compartments.

### 2.5. IL-1β and the Inflammasome Pathway

Similarly to the inflammatory cytokines assessed previously, IL-1β has been studied for is central role in the inflammatory response. This interleukin is engaged in different pathways of the innate immunity. In this article, we wanted to focus specifically on the stimulation of IL-1β via NLRP3 pathway activation. We compared IL-1β and NLRP3 biomarkers in the CSF and serum (Table 6).

Mechanistically, ASC (apoptosis-associated speck-like protein containing a CARD) is the adaptor recruited by the NRLP3 sensor, which once assembled, will recruit and activate Caspase-1. Altogether, this complex becomes active and will, in part, cleave pro-IL-1β and pro-IL-18 into their active forms. In addition to being a downstream biomarker of the NLRP3 pathway, IL-1β acts as an upstream primer as well. Binding of IL-1β to its receptor, IL-1R1, leads to upregulation of the NLRP3 component [36].

Measuring components and biomarkers associated with the pathway, ASC and IL-18, the only markers quantifiable in CSF, were not significantly different in the NIC and MCI cohorts (NIC: 45.20 pg/mL, MCI: 48.83 pg/mL, and NIC: 2.622 pg/mL; MCI: 3.325 pg/mL, respectively).

**Figure 2:**
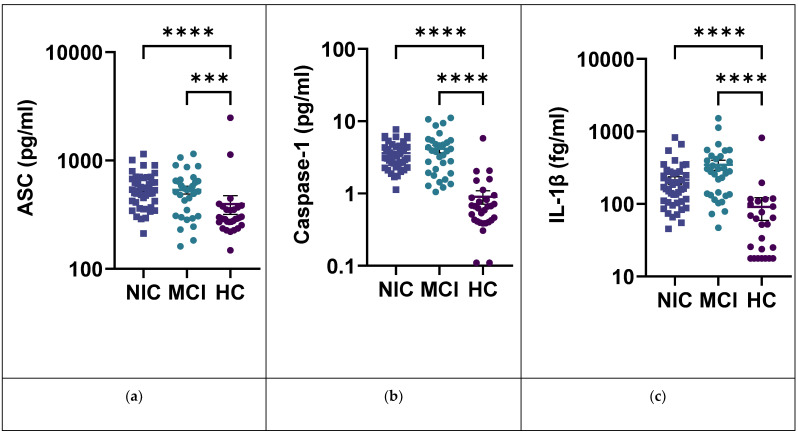
Inflammasome pathway biomarkers. ASC, caspase-1 and IL-β scatter plot. Data are presented as mean (+/−SEM). (**a**) Serum ASC; NIC: 550.0 (29.71) fg/mL, MCI: 535.3 (43.81) fg/mL, and HC: 396.3 (77.82) fg/mL. (**b**) Serum caspase-1; NIC: 3.626 (0.2165) pg/mL, MCI: 4.151 (0.4801) pg/mL, and HC: 0.9008 (0.1915) pg/mL. (**c**) Serum IL-1β; NIC: 211.7 (24.93) fg/mL, MIC 345.2 (54.26) fg/mL, and HC: 90.74 (31.52) fg/mL. ***: *p*-value < 0.001; ****: *p*-value < 0.0001.

Similarly, in serum, no significant difference was observed for any biomarker between the NIC and MCI cohorts. Additionally, serum Caspase-1 and IL-1β had higher levels in the MCI cohort than the NIC cohort but not significantly (Figure 2). Yet all NRLP3 biomarkers were significantly increased in both the NIC and MCI cohorts compared with the HC cohort. Only the IL-18 levels were similar in the three cohorts. Plus, serum Caspase-1, IL-1β, and IL-1Ra had greater heterogeneity in the MCI cohort compared with the other cohorts.

**Figure 3:**
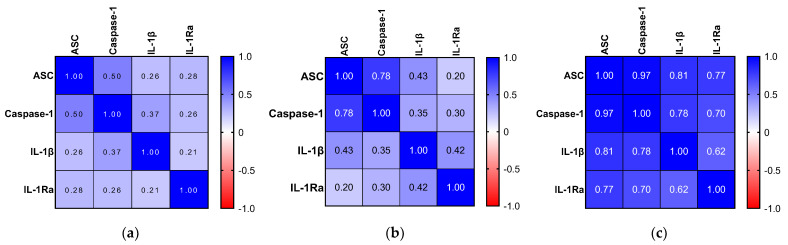
Correlation between NLRP3 serum biomarkers heatmap. Spearman r-value. (**a**) HC cohort. (**b**) NIC cohort. (**c**) MCI cohort. The *p*-value of significance used was < 0.05. In addition, the r-value was required to be ≥ 0.50 or ≤ −0.50 in order to be considered statistically significant.

Comparing correlations between the NLRP3 biomarkers in serum, r-values obtained via the Spearman test were all ≥0.50 and much higher in the MCI cohort (Figure 3, Table S6). Likewise, in this cohort, all biomarkers correlated positively with each other with all *p*-values < 0.0001. In the NIC cohort, only ASC correlated positively with caspase-1 (r = 0.78; *p* < 0.0001). A similar result was observed in the HC cohort, where only ASC and caspase-1 had a positive correlation (r = 0.50; *p* < 0.01).

ASC and caspase-1 are recruited at the very beginning of the cascade, which is demonstrated by the correlations in all three cohorts. However, IL-1β and IL-1Ra are upstream and downstream biomarkers and their link with the NLRP3 complex is not so direct. This was reflected by comparable correlations between the NIC and HC cohorts, whereas it was much stronger in the MCI population. Hence, our results suggest a specific overexpression of the inflammasome pathway in the periphery of MCI patients.

To explore the activation of the pathway in the CNS compartment, we tested correlations between ASC and IL-18, as these two biomarkers are the only ones quantifiable in CSF. In the MCI cohort, there was no significant correlation between the matrix for each biomarker (Table S5). In the NIC population, IL-18 correlated positively in serum vs. CSF (IL-18: r = 0.575, *p* < 0.0001). Hence, the ASC level in the serum is not linked with the ASC level in CSF, and potentially the inflammasome could be activated independently by CNS-secreted cytokines.

**Figure 4:**
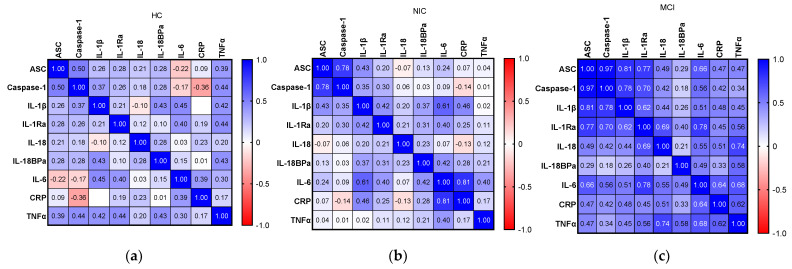
Correlation between NLRP3 and systemic inflammation serum biomarkers heatmap. Spearman r-value. (**a**) HC cohort, (**b**) NIC cohort, and (**c**) MCI cohort.

We added serum inflammatory biomarkers (IL-18, IL-6, CRP, and TNFα) to compare with the serum NRLP3-related biomarkers (Figure 4). Again, in serum, of all three cohorts, stronger correlations were observed in the MCI cohort, with all *p*-values < 0.05, except for caspase-1 vs. TNFα. Interestingly, in the NIC cohort, the correlation between CRP and IL-6 was stronger compared with the MCI and HC cohorts (NIC: r = 0.81, MCI: r = 0.64, and HC: r = 0.39). This confirmed the high peripheral inflammatory status in the NIC cohort, supported by the increased levels obtained for both biomarkers.

Correlations between the NLRP3 biomarkers were comparable between the NIC and HC cohorts, whereas it was much stronger in the MCI population. Our results suggest a specific activation of the inflammasome pathway in the periphery of MCI patients.

### 2.6. Relationship between Inflammatory Biomarkers and AD Hallmarks

Analyzing the NIC and MCI cohorts’ peripheral and central inflammatory statuses, it appeared that CNS inflammation was more specific to AD. Subsequently, we explored interactions between the AD signature and inflammatory processes. We tested the relationships between the hallmark biomarkers (Aβ42, tTau, and pTau181 CSF levels) with all assessed mediators cited above.

Despite previous evidence supporting that peripheral and central inflammatory responses were occurring independently, we first correlated AD biomarkers with all mediator results obtained in serum. No significant results were observed in both cohorts, confirming our first hypothesis.

**Figure 5:**
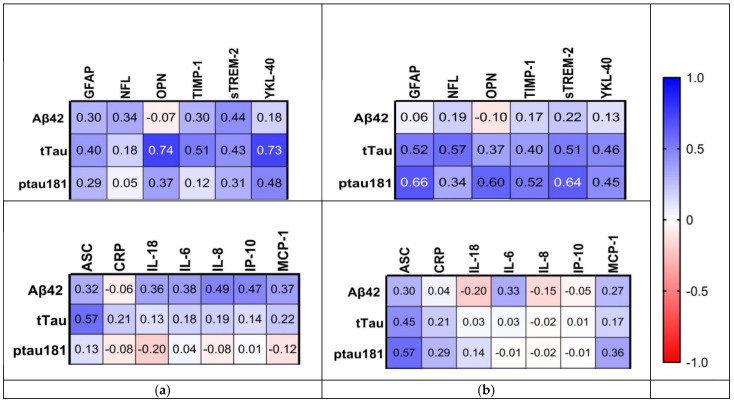
Correlation between AD hallmarks and inflammatory biomarkers. Spearman’s r-value heatmap. (**a**) NIC cohort; (**b**) MCI cohort.

Then, we compared AD hallmarks with neuroinflammation, astrogliosis, and microglia activation biomarkers. The Rr-value results are represented as a heatmap in Figure 5. First, we noticed that there were no correlations between Aβ42 and any markers, underlining its potential limitation in AD diagnosis. On the other hand, we found specific pTau181 correlations in the MCI cohort. The results demonstrated significant correlations between pTau181 and astrocytic and microglial biomarkers, including mediators involved in plaque clearance (OPN, TIMP-1, and sTREM-2). Additionally, pTau181 correlated with the inflammasome biomarker ASC. tTau proteins correlated as well with specific CNS biomarkers in the MCI cohort (GFAP, NFL, and sTREM-2) and the NIC cohort (OPN, TIMP-2, and YKL-40).

To summarize, our results displayed no link between AD hallmarks and peripheral mediators. However, we found a specific pTau181 correlation with central nervous system and inflammasome-related biomarkers in the MCI cohort.

## 3. Discussion

In the present study, we assessed a set of various serum and CSF biomarkers, including AD hallmarks and central nervous system and peripheral system inflammatory mediators, in a cohort of 30 healthy control, 45 non-impaired control, and 30 mild cognitively impaired patients. Our results confirmed specific activation of inflammatory processes in the brain of the MCI cohort. Additionally, the presence of systemic biomarkers in the CSF of the MCI population could give an indication of blood–brain barrier (BBB) permeability. Finally, IL-1β was upregulated in MCI serum and correlated with NLRP3 activation biomarkers.

First, comparing the AD hallmarks in the MCI and NIC cohorts, we were not able to discriminate between the NIC and MCI patients. Aβ42 CSF levels in the MCI cohort were comparable to published data in similar populations and lower in the NIC subjects compared with the MCI population [39,42,43,44]. Nonetheless, both populations had higher Aβ42 CSF levels compared with a demented cohort. The MCI cohort patients are at an early stage of dementia and not AD yet; as a result, it is not surprising that the values in this cohort are higher than mild AD. The cutoffs found in published studies were mainly determined based on AD cohorts; hence, it is difficult to apply them to the MCI cohort, but they can provide clues regarding patients with early AD patterns. Low levels of CSF Aβ42 have been associated with a cognitively normal aged population in addition to being a marker of dementia in AD [45]. Studies demonstrated that postmortem imaging of amyloid depositions did not necessarily correlate with dementia [46]. Furthermore, soluble Aβ42 levels only provide a small clue to the amount of amyloid degradation at a certain timepoint and do not reflect the plaque deposition process. Considering our results, the low Aβ42 levels found in the NIC cohort might not be associated with pathological Aβ deposition, but rather that the levels were too low to allow aggregation in the brain. In addition, the process responsible for plaque clearance might be upregulated in response to the high inflammatory status of the patients. Indeed, Aβ accumulation in Alzheimer patients has been linked to an imbalance between Aβ production and clearance. A study demonstrated that this imbalance is caused by an impaired clearance rate but not by an increased amyloid production [47]. On the opposite, soluble Aβ42 levels in the MCI cohort might reflect an early stage of pre-aggregates. As patients are at the beginning of the disease, the Aβ clearance mechanism could start to gradually slow down and Aβ to slowly and abnormally accumulate before aggregating in the brain. Hence, the first pathological symptoms appear at this stage.

The CSF and serum levels of tTau and pTau181 were not significantly different between the MCI and NIC populations either, but they were significantly higher in serum compared with the HC subjects. Like the Aβ42 results, this could be explained by the fact that the samples were from patients with early onset of disease and that tTau and pTau have also been observed in a normal aging population. Nevertheless, the pTau181/Aβ42 ratio matched with the one found in a published study, confirming that our MCI cohort corresponded to a pre-dementia stage of the disease [40]. The ratio was significantly higher in the NIC cohort compared with the MIC cohort but remained lower than a demented population. Looking strictly at AD hallmark concentrations, both Tau and Aβ42 levels were not sensitive enough, alone or in combination, to diagnose our MCI population. Our results suggest that the NIC population might display more biochemical AD features than the MCI cohort. This highlights a potential lack of specificity for these biomarkers, as amyloidosis and tauopathy are not limited to AD. Moreover, Aβ42 did not correlate with inflammatory cytokines.

AD has been described as a cascade of several biochemical mechanisms [48,49]. First, the amyloid plaques start to accumulate abnormally in the brain, triggering an inflammatory response that will chronically exacerbate amyloid deposition and neurotoxicity. This will be followed by the production and hyperphosphorylation of tau proteins generating neurofibrillary tangles. All three mechanisms together are then responsible for altering neuronal transmission in the brain, which results in cognitive decline. Consequently, it is the combination of amyloids, inflammation, and tau proteins together that is responsible for cognitive impairment. As our patients have MCI based on cognitive tests and potentially early AD onset, the stage of the disease could correspond to the transition between amyloid aggregation and inflammatory response activation. Accordingly, the tauopathy might not be settled at this time. Therefore, tau levels are not yet significantly increased in the MCI cohort compared with the NIC cohort. Furthermore, this highlights a limitation of our study as we are lacking healthy CSF to compare with. Unfortunately, while it is possible to have access to healthy serum, CSF collection is limited to diseased patients only.

In addition, although AD hallmarks could not differentiate between the MCI and the NIC cohorts at this stage, all CNS inflammatory biomarkers, such as the astrogliosis and neuronal damage (GFAP and NFL) were increased in the CSF of the MCI cohort, including significantly for OPN and YKL-40, confirming published results [23,26,28,31,50,51]. More surprisingly, this was not reflected in the serum, where levels were either comparable or increased in the NIC cohort. Given the fact that our NIC patients suffer from osteoarthritis, this cohort can be used as a control for the inflammatory state in systemic circulation. This was confirmed by looking at the blood inflammatory biomarkers. Serum IL-6 and CRP levels were significantly higher in the NIC cohort, supporting published evidence on inflammatory activation in knee arthritis and disc herniation [52,53]. Similarly, IL-1β, IL-8, and TNFα serum levels have been associated with osteoarthritis (OA) [54]. This was demonstrated by our results as concentrations of these biomarkers were comparable in the MCI and NIC cohorts and both significantly increased compared with the HC cohort. Moreover, these results illustrate that peripheral inflammatory biomarkers are hardly specific to AD. Several factors can influence the inflammatory response and activate the production of cytokines independently or in addition to the disease. Furthermore, MCI and AD are age-related diseases and, therefore, the risk of associated inflammatory conditions are exacerbated.

On the other hand, all inflammatory biomarkers tested in CSF where higher in the MCI cohort, with IL-8 and IP-10 levels significantly increased. Altogether, our results support that, in the brain, inflammatory biomarkers are specific to MCI and dementia. Hence, if blood biomarkers could greatly facilitate AD diagnosis, this would require finding a highly specific inflammatory pathway. Evidence from our study suggests that CSF inflammatory biomarkers are more reliable for this neurological disorder. Moreover, the use of blood biomarkers as an appropriate surrogate to CSF remains challenging. Indeed, in our study, there was no significant correlation between CSF and serum for CNS and inflammatory biomarkers in the MCI cohort, whereas some correlations were observed in the NIC cohort. Published data in peri-operative neurocognitive disorder in the context of delirium have also reported unrelated inflammatory cytokines in CSF and serum, suggesting that the CNS inflammatory response might be regulated separately from the peripheral one [55,56]. This could indicate that cytokines may be released by several cell types present in the periphery and in the brain. Considering that two different inflammatory processes are ongoing, determining their time-related interactions could help understand disease onset and progression. Whether these two processes are completely separated or interdependent remains to be investigated. Evidence from the literature supports the role of blood–brain permeability to explain how inflammatory processes from the periphery and CNS are related [57,58,59]. Blood–brain barrier disruption has been investigated in neurodegenerative disease to amplify CNS inflammation via infiltrating cytokines and monocytes [60,61,62]. Mediators from the periphery, once they cross the BBB, might trigger and activate infiltrated monocytes and astroglia [63]. Looking at our biomarkers, CRP plays a control role as the only blood-borne mediator. This protein is produced in response to innate immune cytokines IL-6 and TNFα and is routinely measured in clinical practice as a systemic inflammation biomarker. High CRP blood levels have been associated with neurological disease and depression, but little is known about the relationship between CSF and blood levels [27,64]. In our study, this was particularly striking for CRP, as the MCI cohort had a low serum CRP level, but higher CRP CSF levels compared with the NIC cohort. The same was observed with IL-6, which can be considered a general inflammation marker similar to CRP. Hence, this could reflect potential BBB breakdowns or permeability changes allowing proteins to cross into the CNS. Additionally, IL-8, IP-10, and MCP-1, which are both significantly increased in MCI, have been highlighted to play a role in monocyte infiltration and BBB permeability [31,63,65,66,67]. Indeed, these cytokines can induce chemotaxis of immune cells, and activation and recruitment of microglia to inflammatory sites [68,69,70].

Finally, regarding the IL-1β results, only serum concentration was measurable in our samples as concentrations remained too low to be quantified in CSF. IL-1β concentration was significantly higher in the MCI patients compared with the HC cohort. This difference was also observed in the MCI vs. NIC patients, although not significantly. Additionally, all NLRP3 biomarkers (ASC, caspase-1, and IL-18) correlated positively and significantly with each other and with IL-1β in the MCI population compared with the HC and NIC cohorts. This confirmed the role of the inflammasome pathway and upregulation of IL-1β in pre-dementia patients [38]. An animal model study also demonstrated the potential role of pathological tau to activate IL-1β production via the NLRP3 pathway [71]. Nevertheless, further investigation is needed to comprehend the inflammasome pathway impact and role in the brain. Unfortunately, CSF levels were too low to be detected for most biomarkers of the NRLP3 cascade except for ASC and IL-18, preventing us from drawing any conclusions. However, if protein quantification remains limited in CSF, such as for caspase-1 and IL-1β, we cannot exclude their potential role in central nervous system inflammation. Indeed, focusing on ASC, we observed no correlation between CSF vs. serum in the MCI cohort, suggesting a different origin in the periphery and in the CNS of MCI subjects. Moreover, the results from our study illustrated that ASC correlated significantly with the AD pTau181 hallmark for MCI patients. Moreover, a strong correlation was found in the MCI CSF between ASC and TREM-2, which might indicate activation of the inflammasome pathway through microglia in the brain. Hopefully, rapid development and enhancement of ultra-sensitive quantification methods in the coming years will confirm the role of inflammasome in the CNS.

Overall, we observed a higher variability for most biomarkers in the MCI cohort. This increased heterogeneity can be explained by the fact that MCI encompasses a variety of patients at different stages of an evolving disease. Soluble biomarkers only provide us with a glimpse of all the inflammatory processes involved at a given moment of the disorder setting.

## 4. Materials and Methods

### 4.1. Participants

MIC and NIC prospective samples were acquired from the National Bioservice LLC (NBS, Saint Petersburg, Russia). For the MCI population, patients were required to complete a mini-mental state examination with a total score of 20 to 30, and biochemical measurements of amyloid-40, amyloid-42, Tau, and pTau were conducted via NBS (using MAGPIX Cat. No. HNABTMAG-68K, Merck Millipore, Burlington, VT, USA). Additionally, to be included, a diagnosis of MCI due to AD (stage 2–3) or mild AD based on the National Institute on Aging and Alzheimer’s Association (NIA-AA) criteria and at least a 6-month decline in cognitive function documented in the medical record was required. Patients had to be ≥45 years and ≤90 years. For the NIC population, patients completed a mini-mental state examination with a total score ≥ 29. Furthermore, to be included, patients could not suffer from a chronic neurodegenerative disorder or be older than 55 years old. Associated data, such as collection date, age, gender, ethnicity, comorbidities, and treatment, were acquired for both groups.

HC serum was acquired from the commercial vendor BioreclamationIVT LLC (BIOIVT, Westbury, NY, USA). Patients had to be ≥50 years to age match with the MCI and NIC groups with no comorbidities and/or treatment to be included in the study. Associated data were acquired such as collection date, age, gender, and ethnicity.

### 4.2. Ethical Consent

Written informed consent was obtained for all participants respecting the Declaration of Helsinki and applicable local regulations.

### 4.3. Sample Collection

Blood sera were collected into an SST tube (8.5 mL). After collection, the blood samples sat for 30 min to 1 h to allow blood clots to fully form. Then, blood samples were centrifuged at 2000 rcf for 10 min at room temperature. Sera were aliquoted in 2 mL cryovials and put in a freezer at −80 °C before further analysis.

CSF was collected via lumbar puncture in a 15 mL Falcon tube. To remove blood contamination, samples were centrifuged at 300 rcf for 7 min at +4 °C. The CSF was aliquoted in 2 mL cryovials. An additional 200 µL was aliquoted in a cryovial for MCI individuals and used for Aβ40, Aβ42, Tau, and pTau testing by the vendor. Aliquots were placed in a freezer at −80 °C before further analysis.

### 4.4. CSF and Serum Analyses

Absolute quantification of proteins was obtained using immunoassays with different commercially available kits. Alzheimer’s disease hallmarks were assessed using INNOTEST for Aβ42 and tTau (Cat. No. 81576 and Cat. No. 81572 respectively, FUJIREBIO, Tokyo, Japan) and MSD S-plex for tTau and pTau181 (Cat. No. K151AGPS and Cat. No. K151AGMS respectively, Mesoscale Discovery, Rockville, MD, USA). ASC, caspase-1, IL-10, IL-18, IL-1α, IL-1Ra, IL-6, IL-8, IP-10, MCP-1, OPN, TIMP-1, sTREM-2, and YKL-40 were measured using custom simple-plex kits from Protein Simple (San Jose, TX, USA). GFAP and NFL were assessed with Simoa technology using Neurology 2-plex B (Cat. No. 103520, Quanterix, Billerica, MA, USA). CRP was detected using the DuoSet Human CRP kit (Cat. No DY1707, R&D Systems, Minneapolis, MN, USA). IL-1β and TNFα were quantified with MSD S-plex kits (Cat. No. K151ADSS and Cat. No. K15396S, respectively). Immunoassays were performed on serum and CSF in duplicates using the manufacturer’s instructions. The samples were randomized on the plates and per run. A new aliquot was used for each run to avoid thaw–freeze cycles.

The difference in sample size throughout the study is due to the removal of protein concentrations with a concentration coefficient variation of >30%. Additionally, we excluded CSF and serum protein with a low detection rate (<60%). Our final statistical analyses included 15 proteins that were detected in >60% of CSF and 19 proteins detected in >60% of serum. When the detection rate was >60%, the samples with concentrations below the lowest standard of the calibration curve were included and calculated according to the following formula: calculated concentration = (lower limit of detection)/2 × sample dilution factor.

### 4.5. Statistical Analysis

Data obtained via immunoassays were analyzed with Prism version 9.5.1 (GraphPad Software, La Jolla, CA, USA) and TIBCO Spotfire version 11.4 (TIBCO, Palo Alto, CA, USA).

Study population characteristics (sex, MMSE score, comorbidities, and concomitant disease) were compared using the chi-square test. Aβ42 cutoff comparison was performed with the Fisher exact test.

The normality of the data was tested using the Kolmogorov–Smirnov test. Two-group comparisons were performed using the Welsch *t*-test. Multiple group comparisons were conducted using one way ANOVA followed by the Tukey test. For variables following a non-Gaussian distribution, comparisons were made using the Mann–Whitney *t*-test (two group) or the Kruskal–Wallis test (multiple group) followed by Dunn’s pairwise comparison.

In-group biomarker correlations were performed with the Spearman correlation test. The *p*-value of significance used was < 0.05. In addition to the *p*-value, we fixed an arbitrary r-value cutoff of ±0.50 and only considered correlations with r ≥ 0.50 or ≤−0.50 as statistically significant.

## Figures and Tables

**Table 1:** Study population demographic and clinical characteristics.

	Total *n* = 107	HC *n* = 30	NIC *n* = 45	MCI *n* = 32	*p*-Value
Age, years	64.7 (7.8)	59.6 (6.1)	64.8 (6.5)	69.1 (8.3)	***<0.0001***
Sex (female), *n* (%)	63 (58.9)	15 (50)	28 (62.2)	20 (62.5)	0.0655
MMSE (0–30) *	26.4 (22–30)	-	30 (30–30)]	22 (21.75–24)	***<0.0001***
*Comorbidities*, *n* (%)					
Hypertension	12 (11.2)	-	2 (4.4)	10 (31.3)	***0.014***
Ischemic heart disease	12 (11.2)	-	0 (0.0)	12 (37.5)	***<0.0001***
Diabetes	2 (1.9)	-	0 (0.0)	2 (6.3)	0.0893
*Concomitant disease*, *n* (%)					
Knee arthritis	32 (29.9)	-	32 (71.1)	-	-
Disc herniation	11 (10.3)	-	11 (24.4)	-	*-*

* Median (1st quartile–3rd quartile). *p*-values < 0.05 are given in bold–italic entries. HC: healthy control, NIC: non-impaired control, MCI: mild cognitive impairment, MMSE: mini-mental state examination. Continuous variables are described as means (standard deviation (SD)). *p* values were calculated using *t*-test for continuous variables and Chi-square test for categorical variables.

**Table 2:** Alzheimer biomarkers.

Analyte	HC	NIC	MCI	NIC vs. MCI
	*n* = 30	*n* = 45	*n* = 32	*p*-Value
*CSF*				
Aβ42 IT pg/mL	-	496.3 (235.6)	735.7 (285.1)	***0.0002***
tTau pg/mL	-	211.6 (235.3)	198.1 (186.8)	0.1263
pTau181 pg/mL	-	15.51 (8.149)	16.22 (12.95)	0.3872
*Serum*				
tTau pg/mL	10.01 (12.04)	17.10 (10.87)	13.93 (14.70)	***0.0003*** *
pTau181 pg/mL	-	1.238 (1.989)	0.9307 (1.371)	***0.0173***
*AD cutoffs*, *n* (%)				
Aβ42 < 556 pg/mL	-	28 (62%)	8 (25%)	***0.0023***
*pTau181/Aβ42 ratio*				
pTau181/Aβ42 ^†^	-	0.035	0.018	***0.0008***

* Overall *p*-value from Kruskal–Wallis test between HC, NIC, and MCI. ^†^ pTau/Aβ42 ratio means were calculated using each individual ratio. No CSF available for the HC cohort. Aβ42: amyloid β42; tTau: total Tau; and pTau: phosphorylated tau. *p*-values < 0.05 are given in bold–italic entries.

**Table 3:** Neuroinflammatory biomarkers.

Analyte	HC	NIC	MCI	HC vs. NIC	HC vs. MCI	NIC vs. MCI	Overall ^†^
	*n* = 30	*n* = 45	*n* = 32	*p*-Value	*p*-Value	*p*-Value	*p*-Value
*CSF*							
GFAP pg/mL	-	5737 (3396)	9189 (11,108)	-	-	0.1386	-
NFL pg/mL	-	1164 (1685)	1639 (2086)	-	-	0.0723	-
OPN ng/mL	-	254.8 (119.9)	512.8 (318.0)	-	-	***<0.0001***	-
TIMP-1 ng/mL	-	53.36 (16.96)	63.90 (40.00)	-	-	0.5072	-
sTREM-2 ng/mL	-	14.16 (5.948)	18.05 (10.03)	-	-	0.1332	-
YKL-40 ng/mL	-	172.5 (75.31)	233.6 (123.6)	-	-	***0.0165***	-
*Serum*							
GFAP pg/mL	94.38 (39.56)	133.0 (84.99)	151.8 (132.1)	0.1209	***0.0288***	>0.9999	***0.0272***
NFL pg/mL	14.01 (8.115)	33.67 (29.70)	58.10 (106.5)	***0.0001***	0.2455	0.0699	***0.0002***
OPN ng/mL	41.57 (19.80)	93.07 (42.22)	51.53 (25.25)	***<0.0001***	0.3558	***<0.0001***	***<0.0001***
TIMP-1 ng/mL	111.1 (39.74)	230.6 (58.89)	225.4 (35.75)	***<0.0001***	***<0.0001***	>0.9999	***<0.0001***
sTREM-2 ng/mL	40.49 (19.42)	30.90 (12.31)	28.28 (11.85)	***0.0584***	***0.0088***	>0.9999	***0.0086***
YKL-40 ng/mL	67.05 (69.11)	156.2 (274.9)	66.73 (60.43)	***0.0426***	>0.9999	0.3484	***0.0407***

^†^ Overall *p*-value from Kruskal–Wallis test between the three groups (HC, NIC, and MCI). No CSF was available for the HC cohort. GFAP: glial fibrillary acidic protein. NFL: neurofilament light; OPN: osteopontin; TIMP-1: tissue inhibitor of metalloproteinase 1; sTREM-2: soluble triggering receptor expressed on myeloid cells 2; and YKL-40: Chitinase-3-like 1 (CHI3L1). *p*-values < 0.05 are given in bold–italic entries.

**Table 4:** Systemic inflammatory cytokines in serum.

Analyte	HC	NIC	MCI	HC vs. NIC	HC vs. MCI	NIC vs. MCI	Overall
	*n* = 30	*n* = 45	*n* = 32	*p*-Value	*p*-Value	*p*-Value	*p*-Value
CRP ng/mL	3922 (3604)	12,856 (19798)	3722 (8060)	0.0816	0.0724	***<0.0001***	***<0.0001***
IL-10 pg/mL	2.190 (1.151)	6.094 (5.195)	3.456 (2.120)	***<0.0001***	***0.0086***	***0.0295***	***<0.0001***
IL-6 pg/mL	7.807 (18.71)	42.76 (71.71)	3.162 (3.983)	0.4752	**0.0066**	**<0.0001**	**<0.0001**
IL-8 pg/mL	19.67 (19.65)	35.19 (37.69)	87.74 (164.6)	***0.0054***	***<0.0001***	0.3771	***<0.0001***
IP-10 pg/mL	123.4 (75.34)	97.20 (85.05)	131.7 (79.53)	***0.0238***	>0.9999	***0.0098***	***0.0037***
MCP-1 pg/mL	207.1 (68.54)	536.1 (220.5)	389.6 (155.0)	***<0.0001***	***<0.0001***	***0.0171***	***<0.0001***
TNFα fg/mL	602.7 (249.0)	1592 (648.5)	1235 (447.9)	***<0.0001***	***<0.0001***	0.2253	***<0.0001***

CRP: C-reactive protein; IP-10: interferon gamma-induced protein 10; MCP-1: monocyte chemoattractant protein 1; and TNFα: tumor necrosis factor α. *p*-values < 0.05 are given in bold–italic entries.

**Table 5:** Systemic inflammatory cytokines in CSF.

Analyte	NIC	MCI	NIC vs. MCI
	*n* = 45	*n* = 32	*p*-Value
CRP ng/mL	7.595 (11.18)	11.93 (17.76)	0.8531
IL-6 pg/mL	3. 541 (3.332)	11.63 (25.81)	0.0572
IL-8 pg/mL	41.36 (20.63)	132.8 (198.1)	***0.0002***
IP-10 pg/mL	131.4 (65.38)	199.6 (130.2)	***0.0187***
MCP-1 pg/mL	602.7 (232.4)	671.0 (324.5)	0.3126

IL-8 and IP-10 were the only two biomarkers significantly higher in the MCI cohort CSF compared with the NIC cohort (MCI: 132.8 pg/mL vs. NIC: 41.36 pg/mL, *p* < 0.001; MCI: 131.4 pg/mL vs. NIC: 199.6 pg/mL, *p* < 0.05, respectively). *p*-values < 0.05 are given in bold–italic entries.

**Table 6:** IL-1β and NLRP3 associated biomarkers.

Analyte	HC	NIC	MCI	HC vs. NIC	HC vs. MCI	NIC vs. MCI	Overall
	*n* = 30	*n* = 45	*n* = 32	*p*-Value	*p*-Value	*p*-Value	*p*-Value
*CSF*							
ASC pg/mL	-	45.20 (15.75)	48.83 (26.97)	-	-	0.9243	-
IL-18 pg/mL	-	2.622 (1.830)	3.325 (2.997)	-	-	0.9897	-
*Serum*							
ASC pg/mL	396.3 (426.2)	550.0 (199.3)	535.3 (247.8)	***<0.0001***	***0.0002***	>0.9999	***<0.0001***
Caspase-1 pg/mL	0.9008 (1.049)	3.626 (1.453)	4.151 (2.716)	***<0.0001***	***<0.0001***	>0.9999	***<0.0001***
IL-1β fg/mL	90.74 (157.6)	211.7 (161.5)	345.2 (306.9)	***<0.0001***	***<0.0001***	0.1801	***<0.0001***
IL-1Ra pg/mL	272.3 (164.4)	833.0 (443.2)	684.3 (967.3)	***0.0005***	***0.0177***	0.2925	***0.0007***
IL-18 pg/mL	222.6 (100.1)	223.7 (154.5)	234.0 (114.3)	>0.9999	>0.9999	0.4652	0.3580

No CSF was available for the HC cohort. ASC: apoptosis-associated speck-like protein containing a CARD; IL-1Ra: interleukin-1 receptor antagonist. *p*-values < 0.05 are given in bold–italic entries.

## Data Availability

The data presented in this study are available upon reasonable request from the corresponding author. The data are not publicly available due to a privacy policy.

## Supplementary Materials

The supporting information can be downloaded at: https://www.mdpi.com/article/10.3390/ijms241310523/s1.

## References

[1] Vega J.N., Newhouse P.A. (2014). Mild Cognitive Impairment: Diagnosis, Longitudinal Course, and Emerging Treatments. Curr. Psychiatry Rep..

[2] Mitchell A.J., Shiri-Feshki M. (2009). Rate of Progression of Mild Cognitive Impairment to Dementia—Meta-Analysis of 41 Robust Inception Cohort Studies. Acta Psychiatr. Scand..

[3] Ge X.Y., Cui K., Liu L., Qin Y., Cui J., Han H.J., Luo Y.H., Yu H.M. (2021). Screening and Predicting Progression from High-Risk Mild Cognitive Impairment to Alzheimer’s Disease. Sci. Rep..

[4] (2021). World Alzheimer Report 2021: Journey through the Diagnosis of Dementia.

[5] Winblad B., Amouyel P., Andrieu S., Ballard C., Brayne C., Brodaty H., Cedazo-Minguez A., Dubois B., Edvardsson D., Feldman H. (2016). Defeating Alzheimer’s Disease and Other Dementias: A Priority for European Science and Society. Lancet Neurol..

[6] Alzheimer’s Association (2018). Alzheimer’s Disease Facts and Figures. Alzheimer’s Dement..

[7] Breijyeh Z., Karaman R. (2020). Comprehensive Review on Alzheimer’s Disease: Causes and Treatment. Molecules.

[8] Cummings J.L., Tong G., Ballard C. (2019). Treatment Combinations for Alzheimer’s Disease: Current and Future Pharmacotherapy Options. J. Alzheimer’s Dis..

[9] Cummings J.L., Morstorf T., Zhong K. (2014). Alzheimer’s Disease Drug-Development Pipeline: Few Candidates, Frequent Failures. Alzheimers Res. Ther..

[10] Mullane K., Williams M. (2018). Alzheimer’s Disease (AD) Therapeutics—1: Repeated Clinical Failures Continue to Question the Amyloid Hypothesis of AD and the Current Understanding of AD Causality. Biochem. Pharmacol..

[11] Heneka M.T., O’Banion M.K., Terwel D., Kummer M.P. (2010). Neuroinflammatory Processes in Alzheimer’s Disease. J. Neural Transm..

[12] Heneka M.T., O’Banion M.K. (2007). Inflammatory Processes in Alzheimer’s Disease. J. Neuroimmunol..

[13] Liu C., Cui G., Zhu M., Kang X., Guo H. (2014). Neuroinflammation in Alzheimer’s Disease: Chemokines Produced by Astrocytes and Chemokine Receptors. Int. J. Clin. Exp. Pathol..

[14] Zaheer S., Thangavel R., Wu Y., Khan M.M., Kempuraj D., Zaheer A. (2013). Enhanced Expression of Glia Maturation Factor Correlates with Glial Activation in the Brain of Triple Transgenic Alzheimer’s Disease Mice. Neurochem. Res..

[15] Akiyama H., Barger S., Barnum S., Bradt B., Bauer J., Cole G.M., Cooper N.R., Eikelenboom P., Emmerling M., Fiebich B.L. (2000). Inflammation and Alzheimer’s Disease. Neurobiol. Aging.

[16] Hansson O. (2021). Biomarkers for Neurodegenerative Diseases. Nat. Med..

[17] Aisen P.S., Davis K.L. (2006). Inflammatory Mechanisms in Alzheimer’s Disease: Implications for Therapy. Am. J. Psychiatry.

[18] Liddelow S.A., Guttenplan K.A., Clarke L.E., Bennett F.C., Bohlen C.J., Schirmer L., Bennett M.L., Münch A.E., Chung W.S., Peterson T.C. (2017). Neurotoxic Reactive Astrocytes Are Induced by Activated Microglia. Nature.

[19] Colonna M., Butovsky O. (2017). Microglia Function in the Central Nervous System During Health and Neurodegeneration. Annu. Rev. Immunol..

[20] Jack C.R., Albert M.S., Knopman D.S., McKhann G.M., Sperling R.A., Carrillo M.C., Thies B., Phelps C.H. (2011). Introduction to the Recommendations from the National Institute on Aging-Alzheimer’s Association Workgroups on Diagnostic Guidelines for Alzheimer’s Disease. Alzheimer’s Dement..

[21] Varesi A., Carrara A., Pires V.G., Floris V., Pierella E., Savioli G., Prasad S., Esposito C., Ricevuti G., Chirumbolo S. (2022). Blood-Based Biomarkers for Alzheimer’s Disease Diagnosis and Progression: An Overview. Cells.

[22] Olsson B., Lautner R., Andreasson U., Öhrfelt A., Portelius E., Bjerke M., Hölttä M., Rosén C., Olsson C., Strobel G. (2016). CSF and Blood Biomarkers for the Diagnosis of Alzheimer’s Disease: A Systematic Review and Meta-Analysis. Lancet Neurol..

[23] Mattsson N., Cullen N.C., Andreasson U., Zetterberg H., Blennow K. (2019). Association Between Longitudinal Plasma Neurofilament Light and Neurodegeneration in Patients With Alzheimer Disease. JAMA Neurol..

[24] Weston P.S.J., Poole T., Ryan N.S., Nair A., Liang Y., Macpherson K., Druyeh R., Malone I.B., Ahsan R.L., Pemberton H. (2017). Serum Neurofilament Light in Familial Alzheimer Disease: A Marker of Early Neurodegeneration. Neurology.

[25] Preische O., Schultz S.A., Apel A., Kuhle J., Kaeser S.A., Barro C., Gräber S., Kuder-Buletta E., LaFougere C., Laske C. (2019). Serum Neurofilament Dynamics Predicts Neurodegeneration and Clinical Progression in Presymptomatic Alzheimer’s Disease. Nat. Med..

[26] Milà-Alomà M., Salvadó G., Gispert J.D., Vilor-Tejedor N., Grau-Rivera O., Sala-Vila A., Sánchez-Benavides G., Arenaza-Urquijo E.M., Crous-Bou M., González-de-Echávarri J.M. (2020). Amyloid Beta, Tau, Synaptic, Neurodegeneration, and Glial Biomarkers in the Preclinical Stage of the Alzheimer’s Continuum. Alzheimer’s Dement..

[27] Ng A., Tam W.W., Zhang M.W., Ho C.S., Husain S.F., McIntyre R.S., Ho R.C. (2018). IL-1β, IL-6, TNF- α and CRP in Elderly Patients with Depression or Alzheimer’s Disease: Systematic Review and Meta-Analysis. Sci. Rep..

[28] Van Hulle C., Jonaitis E.M., Betthauser T.J., Batrla R., Wild N., Kollmorgen G., Andreasson U., Okonkwo O., Bendlin B.B., Asthana S. (2021). An Examination of a Novel Multipanel of CSF Biomarkers in the Alzheimer’s Disease Clinical and Pathological Continuum. Alzheimer’s Dement..

[29] Lee W.J., Liao Y.C., Wang Y.F., Lin I.F., Wang S.J., Fuh J.L. (2018). Plasma MCP-1 and Cognitive Decline in Patients with Alzheimer’s Disease and Mild Cognitive Impairment: A Two-Year Follow-up Study. Sci. Rep..

[30] Hu W.T., Howell J.C., Ozturk T., Gangishetti U., Kollhoff A.L., Hatcher-Martin J.M., Anderson A.M., Tyor W.R. (2019). CSF Cytokines in Aging, Multiple Sclerosis, and Dementia. Front. Immunol..

[31] Bettcher B.M., Johnson S.C., Fitch R., Casaletto K.B., Heffernan K.S., Asthana S., Zetterberg H., Blennow K., Carlsson C.M., Neuhaus J. (2018). CSF and Plasma Levels of Inflammation Differentially Relate to CNS Markers of Alzheimer’s Disease Pathology and Neuronal Damage. J. Alzheimers Dis..

[32] Dinarello C.A. (2010). IL-1: Discoveries, Controversies and Future Directions. Eur. J. Immunol..

[33] Testa G., Staurenghi E., Zerbinati C., Gargiulo S., Iuliano L., Giaccone G., Fantò F., Poli G., Leonarduzzi G., Gamba P. (2016). Changes in Brain Oxysterols at Different Stages of Alzheimer’s Disease: Their Involvement in Neuroinflammation. Redox. Biol..

[34] Forlenza O.V., Diniz B.S., Talib L.L., Mendonça V.A., Ojopi E.B., Gattaz W.F., Teixeira A.L. (2009). Increased Serum IL-1β Level in Alzheimer’s Disease and Mild Cognitive Impairment. Dement. Geriatr. Cogn. Disord..

[35] Leung R., Proitsi P., Simmons A., Lunnon K., Güntert A., Kronenberg D., Pritchard M., Tsolaki M., Mecocci P., Kloszewska I. (2013). Inflammatory Proteins in Plasma Are Associated with Severity of Alzheimer’s Disease. PLoS ONE.

[36] Swanson K.V., Deng M., Ting J.P.Y. (2019). The NLRP3 Inflammasome: Molecular Activation and Regulation to Therapeutics. Nat. Rev. Immunol..

[37] Liu L., Chan C. (2014). The Role of Inflammasome in Alzheimer’s Disease. Ageing Res. Rev..

[38] Hanslik K.L., Ulland T.K. (2020). The Role of Microglia and the Nlrp3 Inflammasome in Alzheimer’s Disease. Front. Neurol..

[39] Hulstaert F., Blennow K., Ivanoiu A., Schoonderwaldt H.C., Riemenschneider M., de Deyn P.P., Bancher C., Cras P., Wiltfang J., Mehta P.D. (1999). Improved Discrimination of AD Patients Using β-Amyloid(1-42) and Tau Levels in CSF. Neurology.

[40] van Harten A.C., Wiste H.J., Weigand S.D., Mielke M.M., Kremers W.K., Eichenlaub U., Dyer R.B., Algeciras-Schimnich A., Knopman D.S., Jack C.R. (2022). Detection of Alzheimer’s Disease Amyloid Beta 1-42, p-Tau, and t-Tau Assays. Alzheimer’s Dement..

[41] Gulisano W., Maugeri D., Baltrons M.A., Fà M., Amato A., Palmeri A., D’Adamio L., Grassi C., Devanand D.P., Honig L.S. (2018). Role of Amyloid-β and Tau Proteins in Alzheimer’s Disease: Confuting the Amyloid Cascade. J. Alzheimers Dis..

[42] Bertens D., Tijms B.M., Scheltens P., Teunissen C.E., Visser P.J. (2017). Unbiased Estimates of Cerebrospinal Fluid β-Amyloid 1-42 Cutoffs in a Large Memory Clinic Population. Alzheimers Res. Ther..

[43] Palmqvist S., Zetterberg H., Blennow K., Vestberg S., Andreasson U., Brooks D.J., Owenius R., Hägerström D., Wollmer P., Minthon L. (2014). Accuracy of Brain Amyloid Detection in Clinical Practice Using Cerebrospinal Fluid β-Amyloid 42: A Cross-Validation Study Against Amyloid Positron Emission Tomography. JAMA Neurol..

[44] de Riva V., Galloni E., Marcon M., di Dionisio L., Deluca C., Meligrana L., Bolner A., Perini F. (2014). Analysis of Combined CSF Biomarkers in AD Diagnosis. Clin. Lab..

[45] Mormino E.C. (2014). The Relevance of Beta-Amyloid on Markers of Alzheimer’s Disease in Clinically Normal Individuals and Factors That Influence These Associations. Neuropsychol. Rev..

[46] Perez-Nievas B.G., Stein T.D., Tai H.C., Dols-Icardo O., Scotton T.C., Barroeta-Espar I., Fernandez-Carballo L., De Munain E.L., Perez J., Marquie M. (2013). Dissecting Phenotypic Traits Linked to Human Resilience to Alzheimer’s Pathology. Brain.

[47] Mawuenyega K.G., Sigurdson W., Ovod V., Munsell L., Kasten T., Morris J.C., Yarasheski K.E., Bateman R.J. (2010). Decreased Clearance of CNS Amyloid-β in Alzheimer’s Disease. Science.

[48] Jack C.R., Knopman D.S., Jagust W.J., Shaw L.M., Aisen P.S., Weiner M.W., Petersen R.C., Trojanowski J.Q. (2010). Hypothetical Model of Dynamic Biomarkers of the Alzheimer’s Pathological Cascade. Lancet Neurol..

[49] Huang L.K., Chao S.P., Hu C.J. (2020). Clinical Trials of New Drugs for Alzheimer Disease. J. Biomed. Sci..

[50] Carecchio M., Chiocchetti A., Galimberti D. (2010). Osteopontin Is Increased in the Cerebrospinal Fluid of Patients with Alzheimer’s Disease and Its Levels Correlate with Cognitive Decline. J. Alzheimer’s Dis..

[51] Craig-Schapiro R., Perrin R.J., Roe C.M., Xiong C., Carter D., Cairns N.J., Mintun M.A., Peskind E.R., Li G., Galasko D.R. (2010). YKL-40: A Novel Prognostic Fluid Biomarker for Preclinical Alzheimer’s Disease. Biol. Psychiatry.

[52] Risbud M.V., Shapiro I.M. (2014). Role of Cytokines in Intervertebral Disc Degeneration: Pain and Disc-Content. Nat. Rev. Rheumatol..

[53] Livshits G., Zhai G., Hart D.J., Kato B.S., Wang H., Williams F.M.K., Spector T.D. (2009). Interleukin-6 Is a Significant Predictor of Radiographic Knee Osteoarthritis: The Chingford Study. Arthritis Rheum.

[54] Gundogdu G., Gundogdu K. (2018). A Novel Biomarker in Patients with Knee Osteoarthritis: Adropin. Clin. Rheumatol..

[55] Bromander S., Anckarsäter R., Kristiansson M., Blennow K., Zetterberg H., Anckarsäter H., Wass C.E. (2012). Changes in Serum and Cerebrospinal Fluid Cytokines in Response to Non-Neurological Surgery: An Observational Study. J. Neuroinflamm..

[56] Fertleman M., Pereira C., Dani M., Harris B.H.L., Di Giovannantonio M., Taylor-Robinson S.D. (2022). Cytokine Changes in Cerebrospinal Fluid and Plasma after Emergency Orthopaedic Surgery. Sci. Rep..

[57] Varatharaj A., Galea I. (2017). The Blood-Brain Barrier in Systemic Inflammation. Brain Behav. Immun..

[58] Bettcher B.M., Tansey M.G., Dorothée G., Heneka M.T. (2021). Peripheral and Central Immune System Crosstalk in Alzheimer Disease–A Research Prospectus. Nat. Rev. Neurol..

[59] Prinz M., Priller J. (2017). The Role of Peripheral Immune Cells in the CNS in Steady State and Disease. Nat. Neurosci..

[60] Shang D.S., Yang Y.M., Zhang H., Tian L., Jiang J.S., Dong Y.B., Zhang K., Li B., Zhao W.D., Fang W.G. (2016). Intracerebral GM-CSF Contributes to Transendothelial Monocyte Migration in APP/PS1 Alzheimer’s Disease Mice. J. Cereb. Blood Flow Metab..

[61] Kierdorf K., Masuda T., Jordão M.J.C., Prinz M. (2019). Macrophages at CNS Interfaces: Ontogeny and Function in Health and Disease. Nat. Rev. Neurosci..

[62] Bowman G.L., Dayon L., Kirkland R., Wojcik J., Peyratout G., Severin I.C., Henry H., Oikonomidi A., Migliavacca E., Bacher M. (2018). Blood-Brain Barrier Breakdown, Neuroinflammation, and Cognitive Decline in Older Adults. Alzheimer’s Dement..

[63] Takata F., Nakagawa S., Matsumoto J., Dohgu S. (2021). Blood-Brain Barrier Dysfunction Amplifies the Development of Neuroinflammation: Understanding of Cellular Events in Brain Microvascular Endothelial Cells for Prevention and Treatment of BBB Dysfunction. Front. Cell Neurosci..

[64] Felger J.C., Haroon E., Patel T.A., Goldsmith D.R., Wommack E.C., Woolwine B.J., Le N.A., Feinberg R., Tansey M.G., Miller A.H. (2020). What Does Plasma CRP Tell Us about Peripheral and Central Inflammation in Depression?. Mol. Psychiatry.

[65] Hillmer L., Erhardt E.B., Caprihan A., Adair J.C., Knoefel J.E., Prestopnik J., Thompson J., Hobson S., Rosenberg G.A. (2023). Blood-Brain Barrier Disruption Measured by Albumin Index Correlates with Inflammatory Fluid Biomarkers. J. Cereb. Blood Flow Metab..

[66] Wang K., Wang H., Lou W., Ma L., Li Y., Zhang N., Wang C., Li F., Awais M., Cao S. (2018). IP-10 Promotes Blood–Brain Barrier Damage by Inducing Tumor Necrosis Factor Alpha Production in Japanese Encephalitis. Front. Immunol..

[67] Franciotta D., Martino G., Zardini E., Furlan R., Bergamaschi R., Andreoni L., Cosi V. (2001). Serum and CSF Levels of MCP-1 and IP-10 in Multiple Sclerosis Patients with Acute and Stable Disease and Undergoing Immunomodulatory Therapies. J. Neuroimmunol..

[68] Gertje E.C., Janelidze S., van Westen D., Cullen N., Stomrud E., Palmqvist S., Hansson O., Mattsson-Carlgren N. (2023). Associations Between CSF Markers of Inflammation, White Matter Lesions, and Cognitive Decline in Individuals without Dementia. Neurology.

[69] Michlmayr D., McKimmie C.S. (2014). Role of CXCL10 in Central Nervous System Inflammation. Int. J. Interferon Cytokine Mediat. Res..

[70] Scarpini E., Galimberti D., Baron P., Clerici R., Ronzoni M., Conti G., Scarlato G. (2002). IP-10 and MCP-1 Levels in CSF and Serum from Multiple Sclerosis Patients with Different Clinical Subtypes of the Disease. J. Neurol. Sci..

[71] Jiang S., Maphis N.M., Binder J., Chisholm D., Weston L., Duran W., Peterson C., Zimmerman A., Mandell M.A., Jett S.D. (2021). Proteopathic Tau Primes and Activates Interleukin-1β via Myeloid-Cell-Specific MyD88- and NLRP3-ASC-Inflammasome Pathway. Cell Rep..

