# Supplementary material for: Central and Peripheral Inflammation in Mild Cognitive Impairment in the Context of Alzheimer’s Disease
Source: Int J Mol Sci. 2023 Jun 23;24(13):10523. doi: 10.3390/ijms241310523 (PMC10341890; doi:10.3390/ijms241310523)
Supplement: Supplementary file 1 [file ijms-24-10523-s001.zip › ijms-2419605-supplementary.pdf]

International Journal of
Molecular Sciences

MDPI

Article

Central and Peripheral Inflammation in Mild Cognitive Impairment in the Context of Alzheimer's Disease

Inès Schmidt-Morgenroth [1,2], Philippe Michaud [2], Fabrizio Gasparini [1] and Alexandre Avrameas [1,*]

Supplementary Materials

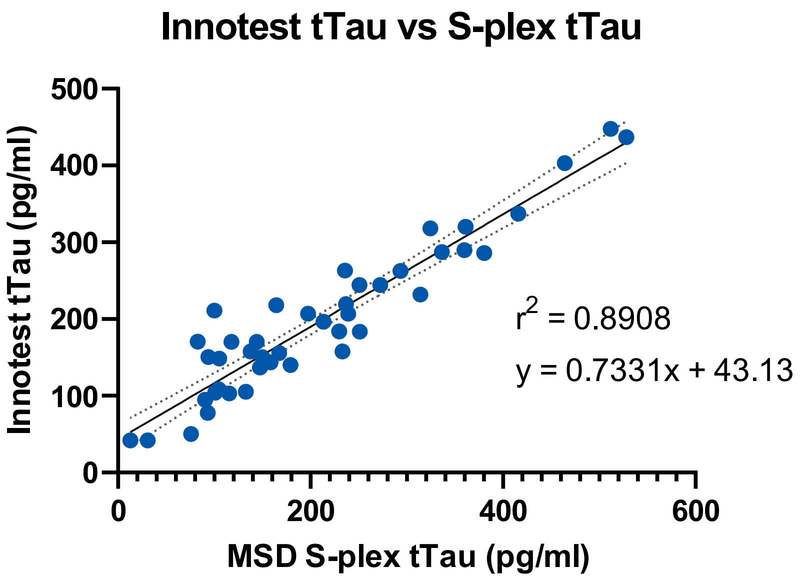

Figure S1. Linear regression between FUJIREBIO INNTOTEST total Tau (tTau) diagnostic test and MESOSCALE DISCOVERY (MSD) S-plex total Tau. Measurements were done with Non-impaired (NIC) CSF samples. Regression curve expression y = 0.7331x + 43.13, r-square = 0.8908 and *p*-value < 0.0001.

(a)

MCI<br>n = 32		CSF biomarkers (r, *p*)		
		**Aβ42**	**tTau**	**pTau181**
**CSF biomarkers** (r, *p*)	**Aβ42**	1.0000<br>-		
	**tTau**	0.2188<br>0.2288	1.0000<br>-	
	**pTau181**	0.0710<br>0.7094	***0.7317***<br>***<0.0001***	1.0000<br>-

(b)

NIC<br>n = 45		CSF biomarkers (r, *p*)		
		**Aβ42**	**tTau**	**pTau181**
**CSF biomarkers** (r, *p*)	**Aβ42**	1.0000<br>-		
	**tTau**	-0.0844<br>0.5858	1.0000<br>-	
	**pTau181**	-0.0762<br>0.6636	***0.5213***<br>***0.0013***	1.0000<br>-

Table S1. Summary table of Spearman correlation between CSF AD hallmarks. r-value and *p*-value are displayed for each correlation as r, *p* respectively. *p*-values < 0.05 and r-values ≥0.50 or ≤-0.50 are given in bold-italic entries. (**a**) Mild Cognitive Impairment (MCI) cohort. (**b**) Non-Impaired control (NIC) cohort.

Aβ: Amyloid β; tTau: total Tau; pTau: phosphorylated tau.

(a)

MCI n = 32		Serum biomarkers (r, *p*) tTau	pTau181
CSF biomarkers (r, *p*)	**Aβ42**	-0.0301	-0.1123
		0.8703	0.5619
	**tTau**	0.2636	0.2064
		0.1450	0.2827
	**pTau181**	0.2169	0.2039
		0.2496	0.3076

(b)

NIC n = 45		Serum biomarkers (r, *p*) tTau	pTau181
CSF biomarkers (r, *p*)	**Aβ42**	-0.0189	-0.0346
		0.9102	0.8388
	**tTau**	0.3805	***0.6195***
		0.0185	***<0.0001***
	**pTau181**	0.3075	***0.5625***
		0.0817	***0.0007***

Table S2. Summary table of Spearman correlation between CSF vs serum AD hallmarks. r-value and *p*-value are displayed for each correlation as r, *p* respectively. *p*-values < 0.05 and r-values ≥0.50 or ≤-0.50 are given in bold-italic entries (a) MCI cohort. (b) NIC cohort.

(a)

MCI n = 32		Serum biomarkers (r, *p*)					
		GFAP	NFL	OPN	TIMP-1	TREM-2	YKL-40
CSF biomarkers (r, *p*)	GFAP	0.1327	0.1411	0.1338	0.0249	0.0374	0.1679
		0.4691	0.4410	0.4654	0.8923	0.8390	0.3584
	NFL	0.2034	0.3083	0.2372	0.0451	-0.1672	0.1309
		0.2641	0.0860	0.1912	0.8064	0.3605	0.4753
	OPN	0.0975	-0.0777	0.1085	0.1246	0.0704	-0.1012
		0.5955	0.6725	0.5544	0.4967	0.7019	0.5817
	TIMP-1	0.2518	0.3691	0.2617	0.2705	0.1507	0.2911
		0.1644	***0.0376***	0.1479	0.1343	0.4105	0.1061
	TREM-2	0.0407	0.0363	0.1536	0.0139	0.0191	0.0916
		0.8250	0.8437	0.4013	0.9397	0.9175	0.6179
	YKL-40	0.2837	0.2991	0.3105	0.2093	0.1393	0.2449
		0.1156	0.0963	0.0837	0.2503	0.4470	0.1768

(b)

NIC n = 45		Serum biomarkers (r, *p*)					
		GFAP	NFL	OPN	TIMP-1	TREM-2	YKL-40
CSF biomarkers (r, *p*)	GFAP	0.4533	0.1891	0.1205	-0.1148	0.2454	0.2221
		0.0023	0.2247	0.4415	0.4637	0.1127	0.1522
	NFL	-0.1323	***0.6498***	0.2884	0.1262	-0.0310	0.0046
		0.3921	***<0.0001***	0.0576	0.4144	0.8416	0.9764
	OPN	0.3290	-0.2163	0.1009	-0.1630	0.1722	0.1801
		0.0273	0.1535	0.5095	0.2848	0.2580	0.2365
	TIMP-1	0.1642	0.2447	0.2868	0.0972	-0.0082	0.1759
		0.2812	0.1053	0.0561	0.5252	0.9575	0.2478
	TREM-2	0.2850	0.1461	0.1132	-0.0584	0.1772	0.0157
		0.0578	0.3382	0.4592	0.7033	0.2442	0.9186
	YKL-40	0.4896	0.0072	0.1665	-0.0568	0.3033	0.3545
		0.0006	0.9623	0.2742	0.7110	***0.0428***	***0.0169***

Table S3. Summary table of Spearman correlation between CSF vs serum neuroinflammatory biomarkers. r-value and *p*-value are displayed for each correlation as r, *p* respectively. *p*-values < 0.05 and r-values ≥0.50 or ≤-0.50 are given in bold-italic entries. (a) MCI cohort. (b) NIC cohort.

GFAP: Glial fibrillary acidic protein. NFL: Neurofilament light; OPN: Osteopontin; TIMP-1: Tissue inhibitor of metalloproteinase 1; sTREM-2: soluble Triggering receptor expressed on myeloid cells 2; YKL-40: Chitinase-3-like 1 (CHI3L1).

(a)

MCI n = 32		Serum biomarkers (r, *p*)					
		**CRP**	**IL-18**	**IL-6**	**IL-8**	**IP-10**	**MCP-1**
CSF biomarkers (r, *p*)	**CRP**	0.1889	0.3962	***0.5009***	0.3452	-0.0734	0.3397
		0.3088	***0.0273***	***0.0041***	0.0617	0.6948	0.0616
	**IL-18**	-0.0844	0.1517	0.2873	0.1262	-0.1139	0.2073
		0.6462	0.4073	0.1108	0.4987	0.5346	0.2549
	**IL-6**	0.2584	0.1378	0.3530	0.3028	-0.0552	0.2518
		0.1533	0.4519	***0.0475***	0.0977	0.7642	0.1644
	**IL-8**	0.1397	0.0356	-0.0561	0.0331	-0.3381	-0.2071
		0.4458	0.8468	0.7602	0.8598	0.0584	0.2554
	**IP-10**	-0.2782	-0.0918	0.1398	0.2194	-0.0919	-0.0106
		0.1231	0.6172	0.4454	0.2358	0.6171	0.9540
	**MCP-1**	0.3727	0.4781	***0.5248***	0.1285	0.1987	0.4202
		0.0357	***0.0056***	***0.0020***	0.4910	0.2757	***0.0167***

(b)

NIC n = 45		Serum biomarkers (r, *p*)					
		**CRP**	**IL-18**	**IL-6**	**IL-8**	**IP-10**	**MCP-1**
CSF biomarkers (r, *p*)	**CRP**	0.3990	0.0848	0.3481	0.4084	0.1329	-0.0042
		0.0098	0.5887	***0.0222***	***0.0065***	0.3956	0.9785
	**IL-18**	-0.1515	***0.6151***	0.0498	0.1765	0.0922	0.1457
		0.3323	***<0.0001***	0.7453	0.2462	0.5471	0.3395
	**IL-6**	0.1630	-0.0052	0.1936	0.3506	0.2905	0.2833
		0.2962	0.9731	0.2025	***0.0182***	0.0529	0.0593
	**IL-8**	-0.1349	0.0020	-0.0559	0.1851	0.3193	-0.0266
		0.3883	0.9894	0.7152	0.2236	***0.0325***	0.8622
	**IP-10**	0.1135	0.1185	0.1948	0.3255	0.3278	0.1262
		0.4687	0.4380	0.1997	***0.0291***	***0.0280***	0.4087
	**MCP-1**	0.1876	0.0061	0.1601	0.3827	0.1591	0.2178
		0.2284	0.9681	0.2935	***0.0095***	0.2965	0.1507

Table S4. Summary table of Spearman correlation between CSF vs serum inflammatory biomarkers. r-value and *p*-value are displayed for each correlation as r, *p* respectively. *p*-values < 0.05 and r-values ≥0.50 or ≤-0.50 are given in bold-italic entries. (a) MCI cohort. (b) NIC cohort.

CRP: C-reactive protein; IL: Interleukin; IP-10: Interferon gamma-induced protein 10; MCP-1: Monocyte chemoattractant protein 1; TNFα: Tumor necrosis factor α.

(a)

**MCI n = 32**		**Serum biomarkers** (r, *p*)	
		**ASC**	**IL-18**
**CSF biomarkers** (r, *p*)	**ASC**	0.1685	0.1327
		0.3566	0.4689
	**IL-18**	0.0322	0.1517
		0.8611	0.4073

(b)

**NIC n = 45**		**Serum biomarkers** (r, *p*)	
		**ASC**	**IL-18**
**CSF biomarkers** (r, *p*)	**ASC**	0.3820	0.1269
		0.0096	0.4062
	**IL-18**	0.1333	***0.5747***
		0.3828	***<0.0001***

Table S5. Summary tables of Spearman correlation between CSF vs serum inflammasome biomarkers. r-value and *p*-value are displayed for each correlation as r, *p* respectively. *p*-values < 0.05 and r-values ≥0.50 or ≤-0.50 are given in bold-italic entries. (a) MCI cohort. (b) NIC cohort. ASC: (apoptosis-associated speck-like protein containing a CARD).

(a)

MCI n = 32		Serum Biomarkers (r, *p*)							
		ASC	Caspase-1	IL-1β	IL-1Ra	IL-18	IL-6	CRP	TNFα
Serum biomarkers (r, *p*)	ASC	1.0000							
		-							
	Caspase-1	***0.9663***	1.0000						
		<0.0001	-						
	IL-1β	***0.8079***	***0.7819***	1.0000					
		<0.0001	***<0.0001***	-					
	IL-1Ra	***0.7735***	***0.7023***	***0.6246***	1.0000				
		<0.0001	***<0.0001***	***<0.0001***	-				
	IL-18	0.4898	0.4223	0.4368	***0.6936***	1.0000			
		0.0044	***0.0160***	***0.0124***	***<0.0001***	-			
	IL-6	***0.6605***	***0.5598***	***0.5111***	***0.7792***	***0.5485***	1.0000		
		<0.0001	***0.0009***	***0.0028***	***<0.0001***	***0.0012***	-		
	CRP	0.4732	0.4172	0.4842	0.4505	***0.5074***	***0.6427***	1.0000	
		0.0062	***0.0175***	***0.0050***	***0.0097***	***0.0030***	***<0.0001***	-	
	TNFα	0.4744	0.3399	0.4517	***0.5562***	***0.7395***	***0.6807***	***0.6197***	1.0000
		0.0093	0.0712	***0.0139***	***0.0017***	***<0.0001***	***<0.0001***	***0.0003***	-

(b)

NIC n = 45		Serum Biomarkers (r, *p*)							
		ASC	Caspase-1	IL-1β	IL-1Ra	IL-18	IL-6	CRP	TNFα
Serum biomarkers (r, *p*)	ASC	1.0000							
		-							
	Caspase-1	***0.7754***	1.0000						
		<0.0001	-						
	IL-1β	0.4325	0.3501	1.0000					
		0.0042	***0.0230***	-					
	IL-1Ra	0.2048	0.2992	0.4221	1.0000				
		0.1773	***0.0459***	***0.0054***	-				
	IL-18	0.0657	0.0561	0.1968	0.2079	1.0000			
		0.6681	0.7145	0.2117	0.1706	-			
	IL-6	0.2372	0.0916	***0.6096***	0.3963	0.0697	1.0000		
		0.1167	0.5494	***<0.0001***	***0.0070***	0.6491	-		
	CRP	0.0671	-0.1440	0.4625	0.2510	-0.1313	***0.8072***	1.0000	
		0.6692	0.3569	***0.0027***	0.1045	0.4013	***<0.0001***	-	
	TNFα	0.0435	0.0104	0.0169	0.1095	0.1196	0.4001	0.1673	1.0000
		0.8260	0.9581	0.9360	0.5792	0.5443	***0.0349***	0.4043	-

(c)

HC n = 30		Serum Biomarkers (r, *p*)							
		ASC	Caspase-1	IL-1β	IL-1Ra	IL-18	IL-6	CRP	TNFα
Serum biomarkers (r, *p*)	ASC	1.0000							
		-							
	Caspase-1	0.4965	1.0000						
		0.0053	-						
	IL-1β	0.2582	0.3733	1.0000					
		0.2126	0.0661	-					
	IL-1Ra	0.2770	0.2629	0.2135	1.0000				
		0.1384	0.1604	0.3055	-				
	IL-18	0.2148	0.1818	0.0961	0.1237	1.0000			
		0.2544	0.3363	0.6478	0.5147	-			
	IL-6	-0.2152	-0.1715	0.4510	0.4045	0.0336	1.0000		
		0.2535	0.3647	***0.0236***	***0.0266***	0.8601	-		
	CRP	0.0857	-0.3633	0.0039	0.1916	0.2294	0.3900	1.0000	
		0.6526	***0.0484***	0.9853	0.3105	0.2226	***0.0331***	-	
	TNFα	0.3944	0.4382	0.4160	0.4398	0.1996	0.3034	0.1665	1.0000
		0.0342	***0.0174***	***0.0386***	***0.0170***	0.2993	0.1095	0.3880	-

Table S6. Summary tables of Spearman correlation between serum inflammasome and inflammatory biomarkers. r-value and *p*-value are displayed for each correlation as r, *p* respectively. *p*-values < 0.05 and r-values ≥0.50 or ≤-0.50 are given in bold-italic entries. (a) MCI cohort. (b) NIC cohort. (c) Healthy Control (HC) cohort. IL-1Ra: Interleukin-1 receptor antagonist.
